# Use of gene sequences as type for naming prokaryotes: Recommendations of the international committee on the taxonomy of chlamydiae

**DOI:** 10.1016/j.nmni.2023.101158

**Published:** 2023-06-21

**Authors:** Gilbert Greub, Trestan Pillonel, Patrik M. Bavoil, Nicole Borel, Lee Ann Campbell, Deborah Dean, Scott Hefty, Matthias Horn, Servaas A. Morré, Scot P. Ouellette, Yvonne Pannekoek, Mirja Puolakkainen, Peter Timms, Raphael Valdivia, Daisy Vanrompay

**Affiliations:** a Centre for Research on Intracellular Bacteria, Institute of Microbiology, University Hospital Centre and University of Lausanne, Bugnon 48, 1011, Lausanne, Switzerland; b Department of Microbial Pathogenesis, University of Maryland School of Dentistry, Baltimore, MD, USA; c Department of Pathobiology, Institute of Veterinary Pathology, Vetsuisse Faculty, University of Zurich, Winterthurerstrasse 268, CH-8057, Zurich, Switzerland; d Department of Global Health, University of Washington, Seattle, WA, USA; e Departments of Medicine and Pediatrics, University of California San Francisco School of Medicine, Oakland, CA, USA; f Department of Molecular Biosciences, University of Kansas, Lawrence, KS, USA; g Centre for Microbiology and Environmental Systems Science, University of Vienna, 1090, Vienna, Austria; h Laboratory of Immunogenetics, Department of Medical Microbiology and Infection Control, VU University Medical Center, Amsterdam, the Netherlands; i Institute for Public Health Genomics (IPHG), Department of Genetics and Cell Biology, Research School GROW (School for Oncology & Developmental Biology), Faculty of Health, Medicine & Life Sciences, University of Maastricht, Maastricht, the Netherlands; j Dutch Chlamydia Trachomatis Reference Laboratory, Department of Medical Microbiology & Infection Control, VU University Medical Centre, Amsterdam, the Netherlands; k Department of Pathology and Microbiology, University of Nebraska Medical Center, Omaha, NE, USA; l Department of Medical Microbiology, Amsterdam UMC, University of Amsterdam, Amsterdam, the Netherlands; m Department of Virology, University of Helsinki and Helsinki University Hospital, Helsinki, Finland; n Genecology Research Center, University of Sunshine Coast, Queensland, Australia; o Department of Molecular Genetics and Microbiology, Duke University, Durham, NC, 27710, USA; p Department of Animal Science and Aquatic Ecology, Faculty of Bioscience Engineering, Ghent University, Coupure Links 653, B-9000, Ghent, Belgium

**Keywords:** SeqCode, Taxogenomics, Chlamydiales, Intracellular bacteria, Chlamydia

## Abstract

The International Committee on Systematics of Prokaryotes (ICSP) discussed and rejected in 2020 a proposal to modify the International Code of Nomenclature of Prokaryotes to allow the use of gene sequences as type for naming prokaryotes. An alternative nomenclatural code, the *Code of Nomenclature of Prokaryotes Described from Sequence Data* (SeqCode), which considers genome sequences as type material for naming species, was published in 2022. Members of the ICSP subcommittee for the taxonomy of the phylum *Chlamydiae* (*Chlamydiota*) consider that the use of gene sequences as type would benefit the taxonomy of microorganisms that are difficult to culture such as the chlamydiae and other strictly intracellular bacteria. We recommend the registration of new names of uncultured prokaryotes in the SeqCode registry.

## 1 Main text

The classification and naming of prokaryotes is essential for communication in biological research. While an increasing number of bacterial taxa are described based on genome sequences only, only cultured bacteria can serve as type material to name new species. The International Committee on Systematics of Prokaryotes (ICSP) held a virtual discussion in 2020 on proposals to use gene/genome sequences as type for the taxonomy of prokaryotes [[1], [2], [3], [4], [5], [6]]. Following the discussion, members of the ICSP voted and rejected these proposals [7], which triggered the development of an alternative nomenclatural code for prokaryotes described from sequence data (SeqCode) [8].

## 2 The requirement for pure culture prevents naming most chlamydiae

Chlamydiologists are facing some difficulties with the current rules of the International Code of Nomenclature of Prokaryotes (ICNP), in particular regarding the deposition of a viable pure culture in two different international collections (rule 30) [9]. We put forth that the proposal to use genome sequences as type [[1], [2], [3]] would in fact be positive for the taxonomy of microorganisms such as members of the chlamydiae that are often very difficult to culture*.* Only a small proportion of all chlamydial species has been described so far [10,11]. As obligate intracellular bacteria that are resistant to axenic growth in pure culture, chlamydiae require cultured eukaryotic cells as host for their own growth. In recent years, an increasing number of *Candidatus* species was described mainly based on genomic data. These include *Candidatus* Chlamydia sanzinia, a snake pathogen [12], *Candidatus* Similichlamydia epinephelii, a fish pathogen [13], *Candidatus* Rhabdochlamydia helvetica, a tick symbiont [14], *Candidatus* Syngnamydia medusae, associated with jellyfish [15] and *Candidatus* Rhabdochlamydia oedothoracis, associated with the dwarf spider *Oedothorax gibbosus* [16]. In fact, our understanding of chlamydial diversity gained a new momentum with the application of single-cell and metagenomic approaches; many of the more recently discovered highly diverse chlamydiae are characterized based on single-cell assembled genomes (SAGs) and metagenome-assembled genomes (MAGs) [[17], [18], [19], [20]]. Our subcommittee thus feels strongly that naming bacterial species based on genomics and metagenomics data should be allowed a) when it has not been possible to isolate the corresponding strain in culture, and b) as long as it is clearly stated that the new strain designation is only based on sequence data. The latter distinction could be achieved by the Whitman and SeqCode proposal of using the “Ts” superscript [3,8] or by continuing to use the *Candidatus* status. If sequence quality standards are applied and appropriate metadata is provided [21], it is possible to establish a consistent taxonomy for the chlamydiae.

## 3 Gene and genome sequences are at the core of prokaryotic taxonomy

While nothing in the ICNP restrict the freedom of taxonomic thought or action (Principle 1), prokaryotic (and eukaryotic) taxonomy heavily rely on gene and genome sequence to build a coherent classification framework [22]. Molecular sequences are more revealing of evolutionary relationships than are classical phenotypes, in particular for prokaryotes [23]. Species are generally delineated using whole genome similarity measures and grouped into higher taxonomic ranks based on phylogenetic analyses [22]. Using sequences as type would facilitate the unambiguous identification of prokaryotic species or even subspecies and prevent the proliferation of synonymous names referring to the same organisms. The genome sequence of Candidatus *Protochlamydia massiliensis,* isolated from *Vermamoeba vermiformis* [24] is for instance nearly identical to that of Candidatus *Protochlamydia naegleriophila* isolated from *Naegleria* amoebae (average nucleotide identity >99%) and should logically be part of the same species [25]. Since both species have a Candidatus status, no priority is officially granted by the ICNP [9]. A centralized registry such as SeqCode with type sequences and associated names would prevent this situation to happen and logically grant priority to the earliest name [8], greatly facilitating the work of taxonomists. Classifying and naming species based on genome sequences would instantly provide an accurate representation of chlamydial diversity. The Genome Taxonomy Database (GTDB) already provides a regularly updated taxonomy of publicly available genomes and could serve as reference for updating the taxonomy of the phylum *Chlamydiae (Chlamydiota*) [26,27].

## 4 The need for quality standards

An important concern is the quality of genome sequences used as type material [6]. Indeed, binning of assembled contigs can lead to errors whereby MAGs are artifactual hybrids of distantly related organism genomes. Evaluation of the quality and completeness of genome assemblies generally rely on the identification of nearly universal marker genes. Tools such as CheckM allow the evaluation of the completeness and potential contamination of any bacterial genome sequence based on the identification of nearly universal bacterial or lineage-specific marker genes [28,29]. The evaluation of the completeness of the genome should be carried out with care considering the large variations in genome size and gene content observed within the phylum. Hybrid assemblies can also be detected by investigating the taxonomic properties of individual genes and contigs with tools such as CAT and BAT [30]. Genome completeness and contamination estimates are already available on GTDB and on the chlamydiae specific database ChlamDB [31]. Some MAGs from poorly characterized chlamydial clades are estimated to be less than 50% complete; however the genome segments that constitute these MAGs can be unequivocally identified as segments of chlamydial genomes [17]. The level of contamination in chlamydial SAGs and MAGs is generally low, and currently only the MAG of *Candidatus* Similichlamydia epinephelii exhibits more than 5% contamination. Phylogenetic analysis indicates that this assembly might represent an admixture of two related strains [17]. Overall, the quality of sequences should not be a major problem if DNA sequences are used as type material for taxonomy of the phylum *Chlamydiae* (*Chlamydiota*).

## 5 Recommendations of the ICSP subcommittee for the taxonomy of the phylum *Chlamydiae* (*Chlamydiota*) to name uncultured species under the SeqCode

The ICSP subcommittee for the taxonomy of the phylum *Chlamydiae* (*Chlamydiota*) consider the SeqCode as a good solution to tackle the problem of naming uncultured prokaryotes. Although the SeqCode is not endorsed by the ICSP [32], we hope that both codes can coexist and be united in the near future. We are in favor of using genomes as type to name uncultured prokaryotes and encourage the use of the “T” and abbreviations to distinguish species with a cultured type (T) from species with a sequence type (Ts). Reference genomes of undescribed species should be registered in the SeqCode registry (https://seqco.de). Data quality standards are discussed in the SeqCode paper [8] and are summarized in Table 1. Raw sequence data and minimal metadata should be associated to the sequence assembly. Reported data should comply with existing standards from the Genomic Standards Consortium [21,33]. The quality of the sequences should be evaluated based on the identification of marker genes and a comprehensive investigation of the taxonomy of individual contigs (e.g. inferred from homology search or phylogenetic analyses [30]). Species should be delineated based on genome average nucleotide identity (ANI) with a cutoff of 95% of identity [2]. The average amino-acid identity (AAI) of orthologous protein sequences can also be used to classify genomes at family (45–65%), genus (65–95%), and species level (95–100%) [2]. Methods based on Percent of Conserved Proteins (POCP) should be used with caution considering the high variability of genome size within the phylum [20]. The classification at higher taxonomic ranks should rely on a phylogeny based on single copy orthologs and the comparison of a set of marker genes such as those used by GTDB [26] or those that have been shown by Pillonel et al. to be taxonomically informative and highly discriminant for the delineation of taxa in the phylum Chlamydiae (*Chlamydiota*) [34]. Genome assemblies of lower quality or those that are incomplete could be used as provisional taxon with the status *Candidatus* and replaced by better quality genome assemblies when available. To improve the consistency of the classification within the phylum, the ICSP subcommittee for the taxonomy of chlamydiae recommends the use of centralized genome sequence databases allowing comparison of new sequences to existing chlamydial genomes and the classification of new genomes in a consistent manner. GTDB with its recently introduced standardized calculation of species clusters and the GTDB Toolkit [26,35], and the chlamydiae-specific genome database ChlamDB [31] are recommended to serve as central resources for the curation of genome-based taxonomy of any members of the phylum *Chlamydiae* (*Chlamydiota*).

**Table 1:** Overview of recommended metadata and quality standards for the use of DNA sequence information to name uncultured taxa. This proposal is partially adapted from Refs. [8,27,37].

Mandatory	Raw sequence data publicly available (e.g. sequencing reads submitted to Sequence Read Archive [SRA])
	Compliance to standards from the Genomic Standards Consortium: provide the minimum information about a genome sequence (MIGS) [34], single amplified genome (MISAG) or metagenome-assembled genome (MIMAG) [21]
	Genome contamination <5% (based on the identification of >100 nearly universal marker genes)
	Genome >90% complete (based on >100 nearly universal marker genes) for species level assignment
	Average amino acid identity (AAI) or whole-genome average nucleotide identity (ANI) to any of the most similar known chlamydial genomes
	Phylogenetic analysis using single copy marker genes and representatives of all known chlamydial genomes [27,28,34]
	For isolates, read coverage≥10x
Recommended	Host name/taxid (if known)
	16*S* rRNA gene(s) sequences >75% complete and passing chimera checks
	High genome integrity (contig no. <100; N50 >25 kilobases (kb); largest contig >100 kb)
	For MAG/SAG, read coverage≥10x
	For MAG/SAG, sequence identified in more than 1 sample (from different places or different time points)

During our previous subcommittee meetings, we recognized the advantages of using genome sequences as type to name members of the phylum *Chlamydiae* (*Chlamydiota*) given its large proportion of yet uncultured representatives [36], and we will continue, within the framework of the ICSP subcommittee and our regular meetings, to work on this important issue. About 40% of all publicly available genome sequences lack a species name [26], indicating that using DNA sequence data as type material will not only facilitate the communication of research on chlamydiae but will be helpful for all Bacteria and Archaea [26]. Thus, the ICSP subcommittee for the taxonomy of the phylum *Chlamydiae* is in favor of using the genomes to name prokaryotes, but with a specific nomenclature, i.e. using the “Ts” epithet.

## Funding information

This work received no specific grant from any funding agency.

## CRediT authorship contribution statement

**Gilbert Greub:** Conceptualization, Writing – review & editing. **Trestan Pillonel:** Conceptualization, Writing – original draft. **Patrik M. Bavoil:** Writing – review & editing. **Nicole Borel:** Writing – review & editing. **Lee Ann Campbell:** Writing – review & editing. **Deborah Dean:** Writing – review & editing. **Scott Hefty:** Writing – review & editing. **Matthias Horn:** Writing – review & editing. **Servaas A. Morré:** Writing – review & editing. **Scot P. Ouellette:** Writing – review & editing. **Yvonne Pannekoek:** Writing – review & editing. **Mirja Puolakkainen:** Writing – review & editing. **Peter Timms:** Writing – review & editing. **Raphael Valdivia:** Writing – review & editing. **Daisy Vanrompay:** Writing – review & editing.

## Declaration of competing interest

The authors declare that they have no known competing financial interests or personal relationships that could have appeared to influence the work reported in this paper.
